# Bottom-up Biomaterial strategies for creating tailored stem cells in regenerative medicine

**DOI:** 10.3389/fbioe.2025.1581292

**Published:** 2025-05-20

**Authors:** Brenda Cruz-Gonzalez, Ellie Johandes, Dominique Gramm, Donny Hanjaya-Putra

**Affiliations:** ^1^ Department of Aerospace and Mechanical Engineering, Bioengineering Graduate Program, University of Notre Dame, Notre Dame, IN, United States; ^2^ Harper Cancer Research Institute, University of Notre Dame, Notre Dame, IN, United States; ^3^ Department of Chemical and Biomolecular Engineering, University of Notre Dame, Notre Dame, IN, United States; ^4^ Center for Stem Cells and Regenerative Medicine, University of Notre Dame, Notre Dame, IN, United States

**Keywords:** biomaterials, 3D printing, stem cells, tissue engineerings, disease modeling, drug screening, nanoparticles, backpack molecules

## Abstract

Biomaterial-assisted stem cell therapies hold immense promise for regenerative medicine, yet clinical translation remains challenging. This review focuses on recent advances and persistent limitations in applying induced pluripotent stem cells (iPSCs), endothelial colony-forming cells (ECFCs), multipotent mesenchymal stromal cells (MSCs), and embryonic stem cells (ESCs) within engineered microenvironments. We introduce a novel “bottom-up” approach to biomaterial design. This approach focuses first on understanding the fundamental biological properties and microenvironmental needs of stem cells, then engineering cell-instructive biomaterials to support them. Unlike conventional methods that adapt cells to pre-existing materials, this strategy prioritizes designing biomaterials from the molecular level upward to address key challenges, including differentiation variability, incomplete matching of iPSCs to somatic counterparts, functional maturity of derived cells, and survival of ECFCs/MSCs in therapeutic niches. By replicating lineage-specific mechanical, chemical, and spatial cues, these tailored biomaterials enhance differentiation fidelity, reprogramming efficiency, and functional integration. This paradigm shift from passive scaffolds to dynamic, cell-instructive platforms bridges critical gaps between laboratory success and clinical translation, offering a transformative roadmap for regenerative medicine and tissue engineering.

## Graphical Abstract

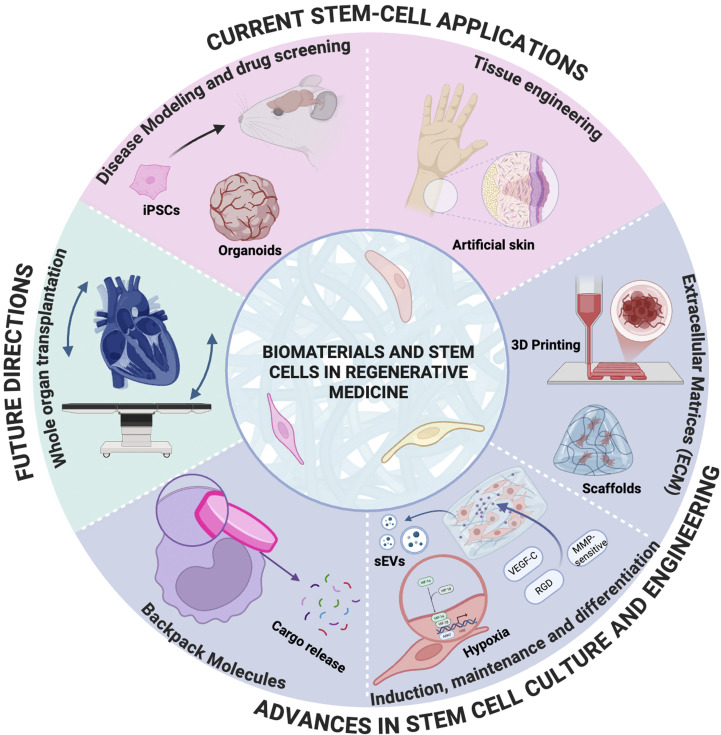

## 1 Introduction

The distinctive capacity of stem cells to self-renew and differentiate positions them as a cornerstone of regenerative medicine and tissue engineering. These properties have enabled breakthroughs in organ-on-a-chip models, organoids, bone grafts, and exosome-based therapies. Recent advances in biomaterial-assisted technologies—such as 3D bioprinting, engineered scaffolds, and spatially controlled microenvironments—are accelerating the clinical translation of stem cell therapies by addressing critical barriers in cell survival, differentiation, and functional integration (Vunjak-Novakovic and Scadden, 2011). However, persistent challenges, including post-implantation teratomas, immune rejection, differentiation variability, and the incomplete functional maturity of derived cells continue to hinder clinical progress. This review focuses on biomaterial-assisted stem cell therapies, emphasizing the innovative use of engineered materials to overcome these limitations. Unlike conventional approaches that adapt stem cells to pre-existing biomaterials, we propose a “bottom-up” design framework where “bottom” refers to the fundamental biological and microenvironmental needs of stem cells (e.g., mechanical cues, biochemical gradients, cell-cell interactions), and “up” represents the development of cell-instructive biomaterials tailored to these requirements. By prioritizing stem cell biology in material design, this strategy addresses key challenges such as differentiation fidelity, functional maturation of embryonic and induced pluripotent stem cells (ESCs and iPSCs), and the survival of therapeutic populations like endothelial colony-forming cells (ECFCs) and mesenchymal stromal cells (MSCs) in hostile microenvironments.

We critically analyze recent advances in biomaterial-driven stem cell culture, differentiation protocols, and clinical applications, highlighting how tailored materials can replicate lineage-specific cues to enhance therapeutic outcomes. This paradigm shift from passive scaffolds to dynamic, cell-instructive platforms offers a transformative roadmap for bridging the gap between laboratory innovation and clinical translation, enabling safer, more effective regenerative therapies.

## 2 A brief overview of stem cells

There are multiple classifications of stem cells based on their origin, differentiation capacity, and function. Here, we summarize the broadest categories of stem cells: Embryonic Stem Cells (ESCs), Somatic Stem Cells (SSCs), and Induced Pluripotent Stem Cells (iPSCs). Additional coverage is also given to SSCs that are widely used in regenerative medicine research, including Mesenchymal Stromal Cells (MSCs) and Endothelial Colony-Forming Cells (ECFCs).

### 2.1 Embryonic stem cells

Embryonic Stem Cells (ESCs) are pluripotent cells that arise from the inner cell mass of an embryo at the blastocyst stage of development, 4–7 days post-fertilization. ESCs then differentiate into the endoderm, mesoderm, and endoderm, giving rise to all somatic cell types in the body (Thomson et al., 1998; National Academies Press US, 2002). As harvesting human ESCs requires the destruction of the embryo, their use in research and medicine is controversial. Prior to 2009, the United States prohibited federal funding to ESC lines generated before 9 August 2001, effectively curtailing the making of new ESC lines. Although these restrictions have somewhat been lifted [see (Matthews and Morali, 2022) for a recent review of laws in the United States governing embryonic research], the ethical and legal dilemmas of using ESCs have caused researchers to seek alternatives (Snead, 2005).

### 2.2 Induced pluripotent stem cells

In 2006, Takahashi et al. identified four transcription factors, Oct4, Sox2, Klf4, and c-Myc, that, when expressed in adult somatic cells, allows them to be reprogrammed into a pluripotent state. These cells, called induced Pluripotent Stem Cells (iPSCs), express ESC markers and have the same capacity for self-renewal and differentiation (Takahashi and Yamanaka, 2006; Takahashi et al., 2007). This breakthrough revolutionized the field of regenerative medicine, opening new avenues for research and therapeutic applications without the ethical concerns of ESCs or the difficulty of sourcing SSCs.

Although iPSCs are a powerful tool for research [see Section 3], there are still roadblocks to the goal of having autologous, iPSC-derived cells available for patients. Although iPSCs have similar morphology and function to ESCs, they still retain epigenetic “memory” from their original phenotype (Kim et al., 2010). This can cause challenges during differentiation, as iPSCs can be biased towards their original lineage (Polo et al., 2010; Bar-Nur et al., 2011). Due to this, donor viability, tissue of origin, as well as differentiation method can influence the reprogramming of iPSCs. According to Kyttala et al., the epigenetic background of the donor highly affects the reprogramming of the iPSCs. While the iPSCs derived from a single source donor are highly similar to each other, genetic differences between donors influence iPSC gene expression patterns and DNA methylation profiles (Kyttälä et al., 2016).

Furthermore, a major concern with iPSCs is their tumorigenic potential. If differentiation is incomplete or residual pluripotent cells persist, iPSCs can form teratomas—tumors containing multiple tissue types (Griscelli et al., 2012). This poses a significant safety challenge, as uncontrolled cell proliferation and incomplete or heterogeneous differentiation can result in unintended tissue formation, thereby limiting their clinical utility. To address these risks, researchers are actively developing and optimizing protocols to ensure complete and precise differentiation of iPSCs before they are used in research or therapeutic applications (Lin et al., 2024; Singh et al., 2015).

Despite current limitations, iPSCs remain an attractive technology for regenerative medicine, drug discovery, and personalized cell therapy, in hopes to tackle neurodegenerative diseases, diabetes, cardiovascular disorders, and cancer (Cerneckis et al., 2024; Rezza et al., 2014).

### 2.3 Somatic stem cells

Somatic Stem Cells (SSCs), also known as Adult Stem Cells (ASCs) are multi and unipotent stem cells found in the adult body. They reside in specialized environments known as stem cell niches. The stem cell niche is composed of the SSCs and their surrounding stromal cells and extracellular matrix (ECM), which provide a combination of mechanical feedback and signaling molecules that maintain the SSCs in a quiescent state. These signals are further influenced by surrounding vasculature, immune cells, and neurons, depending on the SSC lineage (Rezza et al., 2014). The stem cell niche can change to facilitate SSC activation, prompting SSCs to self-renew and differentiate to provide new cells for tissue maintenance and repair (Montagnani et al., 2016; Mannino et al., 2022).

SSCs are found in almost every tissue in the body, including the brain, muscles, fat, bone, intestines, liver, and skin (Brunet et al., 2023). One of the best-characterized SSC is the hematopoietic stem cell (HSC), which is found in the bone marrow. HSCs constantly generate new red blood cells, platelets, and immune cells (Bryder et al., 2006). Bone marrow transplants and hematopoietic cells from umbilical cord blood are used as treatments for patients with blood cancer (Author Anonymous, 2023). With the exception of MSCs [see Section 2.3.1], there are currently no other SSCs that have been approved by the United States Food and Drug Administration (FDA) for medical use.

#### 2.3.1 Multipotent mesenchymal stromal cells

Multipotent Mesenchymal Stromal Cells (MSCs), sometimes referred to as Mesenchymal Stem Cells, are plastic-adherent cells that can differentiate into osteoblasts (bone-forming), adipocytes (fat-forming), and chondrocytes (cartilage-forming) *in vitro* (Mai et al., 2023; Horwitz et al., 2005). However, due to having limited self-renewal, as well as difficulties replicating their differentiation *in vivo*, there is significant debate as to whether or not they can truly be considered a multipotent stem cell (Singh et al., 2015; Caplan, 2017; Phinney et al., 2023).

MSCs are used in regenerative medicine due to their ability to influence the behavior of other cells. They produce a wide range of extracellular vesicles, growth factors, proteins, cytokines, and chemokines, the total of which are referred to as the MSC secretome (Eleuteri and Fierabracci, 2019). MSCs and their secretome modulate immune responses (Dabrowska et al., 2021) and have been shown to promote wound healing, vascularization, and tissue repair, making them an increasingly popular addition in engineered tissues as well as candidate for clinical treatments (Liu et al., 2023a; An et al., 2018; Deng et al., 2020; Wang et al., 2021; Neef et al., 2022; Takeuchi et al., 2021). In December 2024, the first MSC clinical product, Mesoblast’s RYONCIL™, was approved by the FDA to treat Steroid-Refractory Acute Graft-Versus-Host Disease. This disease is a serious and sometimes deadly complication of allogeneic hematopoietic stem cell transplants, during which the transplanted cells attack the recipient’s tissues (Justiz Vaillant et al., 2025). During treatment, MSCs are given via infusion to downregulate inflammatory cytokines while promoting anti-inflammatory cytokine and immune cell activity (Commissioner O of the FDA, 2024; Mesoblast, 2025).

#### 2.3.2 Endothelial colony-forming cells

In 2004, Ingram et al. discovered putative endothelial progenitor cells (EPCs) with robust vascular sprouting ability (Ingram et al., 2004). These cells were previously known by various terms such as late EPCs, large EPCs, and non-hematopoietic EPCs, among others. They are now termed endothelial colony-forming cells (ECFCs). These multipotent cells can give rise to endothelial cells and have the potential to regenerate blood vessels (Ingram et al., 2004). The ability of ECFCs to form vasculature has applications for treating ischemia after heart attack or stroke. For instance, studies show that when ECFCs are introduced into ischemic tissues, they can contribute to neovascularization, improving blood flow and tissue viability (Liu et al., 2024a), (O'Neill et al., 2018). In xenograft models, where human ECFCs are implanted into immunocompromised mice, they have been shown to integrate into the host vasculature and promote angiogenesis. This integration is crucial for restoring blood supply to ischemic tissues, as seen in models of hindlimb ischemia and myocardial infarction (Bryder et al., 2006; Author Anonymous, 2023; Mai et al., 2023; Viswanathan et al., 2019; Dabrowska et al., 2021; Liu et al., 2023a; An et al., 2018).

The therapeutic effects of ECFCs are attributed not only to their ability to differentiate into endothelial cells but also to their secretion of pro-angiogenic factors that stimulate surrounding host cells in ischemic environments through multiple endogenous and exogenous mechanisms that enhance their paracrine activity and therapeutic effects (Caplan, 2017; Deng et al., 2020; Wang et al., 2021). This paracrine signaling enhances the overall angiogenic response in the ischemic environment.

**FIGURE 1:**
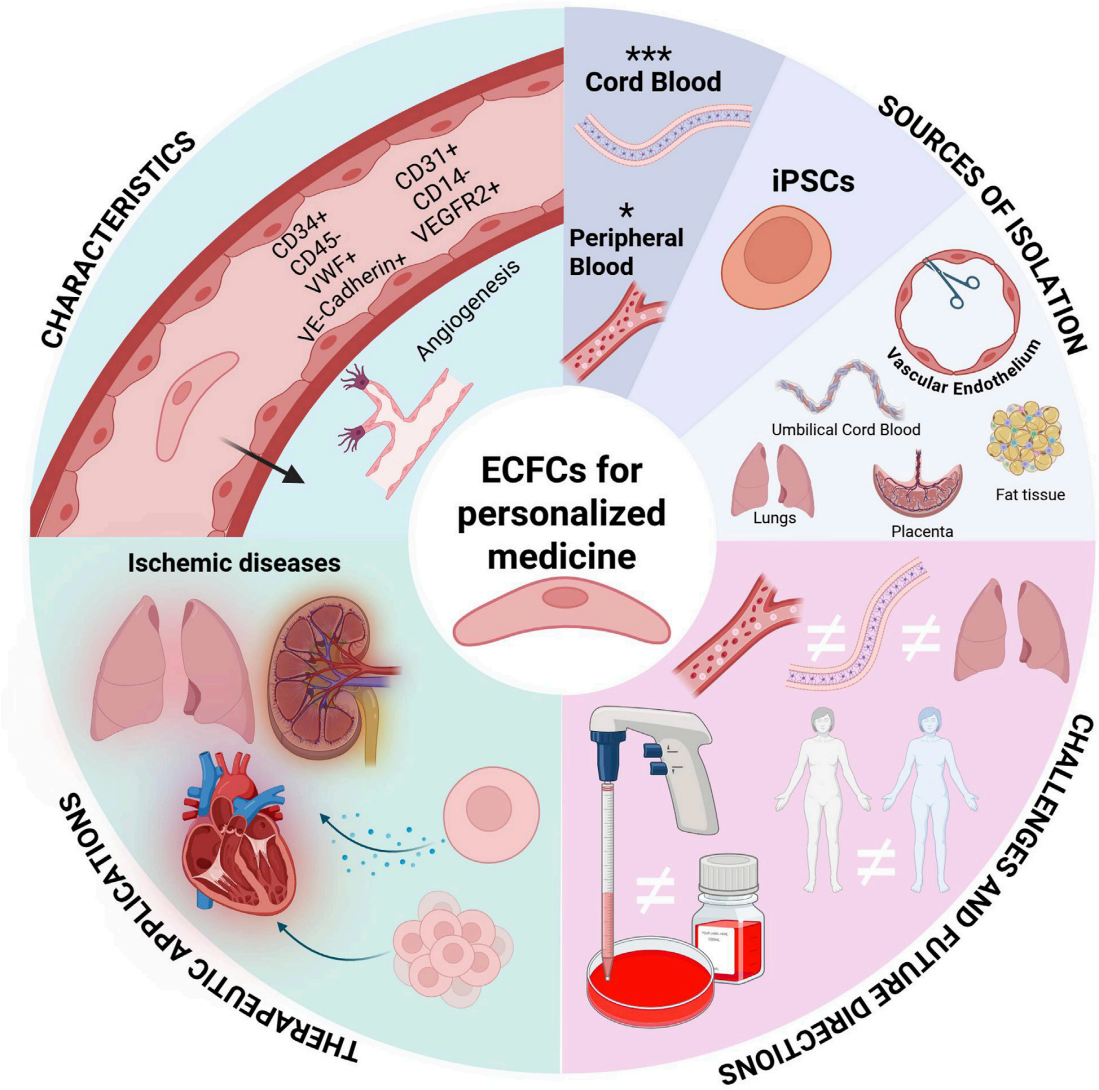
Engineering Endothelial Colony-Forming Cells (ECFCs) for Personalized Medicine. ECFCs have a multifaceted role in personalized medicine. This graphic provides an overview of ECFC characterization, isolation, current challenges, and therapeutic applications.

ECFCs can be derived from a patient’s own peripheral blood, allowing for personalized therapeutic approaches. This method reduces the risk of immune rejection and eliminates the need for immunosuppressive drugs, which are often necessary in allogeneic therapies. There is preclinical evidence that has demonstrated that ECFC administration can enhance vascular stability and promote regeneration of damaged tissues through both direct engraftment and paracrine signaling mechanisms. This highlights their role not only in direct treatment but also in supporting the body’s natural repair processes (Hanjaya-Putra et al., 2013; Hall et al., 2023; Liu et al., 2024b). However, it is noted that ECFCs from non-healthy patients may exhibit dysfunction (Bui et al., 2022; Hanjaya-Putra et al., 2013; Besnier et al., 2021; Melero-Martin, 2022; Hall et al., 2023) necessitating strategies to enhance their functionality before therapeutic application. Furthermore, the media and extracellular matrix coating used to expand isolated ECFC *in vitro* can influence their functionality (Hall et al., 2023; Liu et al., 2024b). Therefore standardized methods for isolating and expanding ECFCs *in vitro* before transplantation are needed (Bell et al., 2023; Varberg et al., 2018). While challenges such as donor-specific dysfunction and expansion protocol variability remain, the unparalleled regenerative capacity of ECFCs Figure 1. —coupled with emerging bioengineering strategies to optimize their function—positions these cells as transformative agents in vascular repair, warranting continued investment to harness their full therapeutic potential.

## 3 Current stem cell applications

### 3.1 Disease modeling and drug screening

Currently, bringing a new drug to the clinical market takes 10–15 years costs approximately $1-2 billion. Much of this time and cost is due to a 90% failure rate during clinical trials (Dowden and Munro, 2019). When looking at failed drugs that initially passed clinical trials, 40% are removed due to low efficacy (Sun et al., 2022); what works on a Petri dish or a mouse may not function similarly for a human being (Tang et al., 2022a; Mozneb et al., 2024). As such, it is paramount to develop more effective preclinical models that capture complex disease behaviors, drug metabolomics, and allow for high-throughput testing. Due to their ability to be passaged almost indefinitely, as well as their ability to differentiate into difficult-to-source cell types, stem cells are now at the forefront of disease modeling.

#### 3.1.1 iPSC disease-specific modeling

There are multiple benefits to using iPSC-derived models in disease research. The first is their ability to differentiate into difficult-to-source human cells, such as neurons and cardiomyocytes. In this role, they can act as a positive control against genetically and chemically-induced models of diseases. For example, a 2019 study of Sporadic Alzheimer’s Disease (SAD) used iPSCs generated from donors with SAD and healthy individuals to generate iPSC-derived neural progenitor cells. These were compared with gene-edited cells to study the causes of neural gene network disruption (Meyer et al., 2019). iPSC-derived cells can also be used to overcome limitations in current mouse models for neurodevelopmental and psychiatric disorders (Deng, 2017; Guerreiro and Maciel, 2023). Furthermore, a single iPSC line can be differentiated into several cell types to generate complex *in vitro* models (e.g., creating a co-culture of both neurons and supporting glial cells) as well as screening multiple cell types for off-target effects in drug research. This makes them suitable for organ-on-a-chip models, which have gained popularity due to their ability to model complex systems and perform high-throughput drug screening in a resource-efficient manner. For instance, iPSCs have been incorporated into chip models of the blood-retinal barrier (ARIK et al., 2021), intestine (Moerkens et al., 2024), heart (Tang et al., 2022a; Mozneb et al., 2024; Ronaldson-Bouchard et al., 2022), and liver (Scheidecker et al., 2024).

Another advantage of iPSCs is their genetic heterogeneity. Factors such as ethnicity, sex, and age impact a patient’s response to medications (Johnson, 2008; Zucker and Prendergast, 2020; Mangoni and Jackson, 2004). For *in vitro* drug screening, increasing the diversity of iPSC patient-donors provides a more robust method for testing the toxicity and functionality of the treatment. Heterogeneity is also critical when searching for the underlying causes of disease. In addition to using cells from “healthy” donors, it is possible to derive iPSCs from patients diagnosed with sporadic and familial diseases (Jang et al., 2012). As such, researchers can compare genotypes across ranges of disease severity, as well as examine diseases which may have several different mutations that can result in the same clinical presentation, such as in cardiovascular diseases (Kelly and Semsarian, 2009; Saha et al., 2024).

To increase the accessibility of diverse iPSC lines in research, there are currently over a dozen well-established stem cell banks worldwide which encompass thousands of cell lines and diseases (Chen et al., 2021; Huang et al., 2019). These include the European Bank for induced pluripotent Stem Cells (EBiSC), California Institute for Regenerative Medicine (CIRM), Fujifilm Cellular Dynamics International (FCDI), and the Taiwan Human Disease iPSC Consortium. Additionally, there exist disease-specific iPSC banks which focus on Sickle-Cell Disease (Park et al., 2017), psychiatric disorders (Rademaker et al., 2018), and aging (Dowrey et al., 2025).

However, iPSC heterogeneity can also originate from differentiation protocols and culture conditions. This is illustrated by Le Cann et al.’s comparison of two differentiation methods in an iPSC-derived model of Huntington Disease. Despite starting from the same iPSC line, the iPSC-derived striatal medium spiny neurons showed differences in voltage-dependent activation and inactivation, as well as protein markers, depending on which differentiation protocol was used (Le Cann et al., 2021). Such differences cast doubt on the validity of iPSC disease models, as seen in the high variability found in a meta-analysis of iPSC Cystic Fibrosis models (Darwish et al., 2022). To ameliorate this problem, researchers could include multiple donors or study iPSC-derived cell models alongside native cells.

Another solution is the establishment of robust negative controls to isolate disease-specific phenotypes from confounding genetic and environmental variables. While early iPSC studies relied on unrelated healthy donor lines as controls, this approach introduced significant heterogeneity due to differences in genetic backgrounds, epigenetic memory, and differentiation biases (Yang et al., 2020; Rauth et al., 2021). Modern strategies prioritize isogenic controls—CRISPR-edited lines derived from the same parental iPSC—to create genetically matched pairs differing only in the disease-causing mutation. Recent protocols combining p53 inhibition and pro-survival small molecules achieve >90% homologous recombination efficiency, enabling rapid generation of isogenic pairs with minimal off-target effects (Singh et al., 2024). For example, in cardiac long QT syndrome models, CRISPR-edited iPSC-derived cardiomyocytes with KCNQ1/KCNH2 mutations showed prolonged action potential durations compared to unedited controls, establishing a template for drug screening (Doss and Sachinidis, 2019).

Beyond genetic standardization, biomaterial platforms can also enhance control validity by recreating tissue-specific physical niches. Hydrogels provide critical standardization for iPSC-derived Parkinson’s Disease (PD) models by recreating brain-specific microenvironments. For example, hyaluronic acid (HA)-based hydrogels have been used to generate midbrain-mimetic 3D cultures, enabling the differentiation of iPSCs into dopaminergic (DA) neurons with forebrain, midbrain, and hindbrain gene expression profiles. These HA scaffolds support organoid development and physiological neurobehavior, offering a biomaterial baseline for comparing healthy and PD-specific phenotypes (Zhu et al., 2025). Similarly, collagen (COLL) hydrogels loaded with glial cell-derived neurotrophic factor (GDNF) enhance the survival and striatal innervation of DA neurons in PD models, providing a functional benchmark for assessing disease-driven connectivity defects (Zhu et al., 2025). These biomaterial frameworks disentangle genetic effects from mechanical confounders, enabling reliable drug screens targeting pathogenic pathways. Finally, emerging best practices validate isogenic controls across multi-omic layers—single-cell transcriptomics to confirm differentiation fidelity, electrophysiological profiling for functional benchmarking, and proteomics to identify off-target editing effects (McTague et al., 2021; Volpato and Webber, 2020). Challenges remain in modeling complex diseases, where residual epigenetic memory in iPSC-derived ‘healthy’ controls may skew results, necessitating validation against primary tissue samples.

#### 3.1.2 Organoids

Organoids are three dimensional clusters of cells cultured *in-vitro* that can self-organize and differentiate into functional cell types (Corrò et al., 2020). The most prominent applications of human organoids lie in their ability to serve as sophisticated models for understanding human diseases, discovering and testing new drugs, and ultimately guiding personalized therapeutic strategies (Yang et al., 2020). Their human origin and 3D architecture provide significant advantages over traditional 2D cell cultures and animal models in biomedical research.

Organoid generation techniques rely on cell sources such as pluripotent stem cells (PSCs), adult stem cells (ASCs), or patient-derived tissues, each selected based on the target organ and research objectives (Calà et al., 2023; Tang et al., 2022b). For example, intestinal organoids require Wnt agonists (e.g., R-spondin) and Noggin to maintain crypt-like structures, while lung organoids depend on FGF7/FGF10 for morphogenesis (Tang et al., 2022b; Unagolla and Jayasuriya, 2022). Culture techniques such as the use of bioreactors also enhance scalability and maturation by improving nutrient/oxygen diffusion through constant spinning, enabling long-term cultures (Calà et al., 2023; Gunti et al., 2021).

Advanced platforms like organoid-on-chip integrate microfluidics to simulate dynamic physiological forces (e.g., shear stress) and automate drug screening (Reumann et al., 2023; Lorenzo-Martín et al., 2024a).

A foundational application of organoids in biotechnology lies in genetic engineering, where they serve as dynamic platforms to model human diseases and dissect gene function with unprecedented precision. CRISPR/Cas9 is widely used in organoid research to model diseases and dissect gene function (Gopal et al., 2020). Early application in pluripotent stem cells (PSCs) or adult stem cells (ASCs) allows precise introduction of mutations. (e.g., APC or TP53 knockouts in colorectal organoids to mimic tumorigenesis) or correction of disease-causing variants (e.g., CFTR repair in cystic fibrosis models) (Gunti et al., 2021; Artegiani et al., 2020)

**FIGURE 2:**
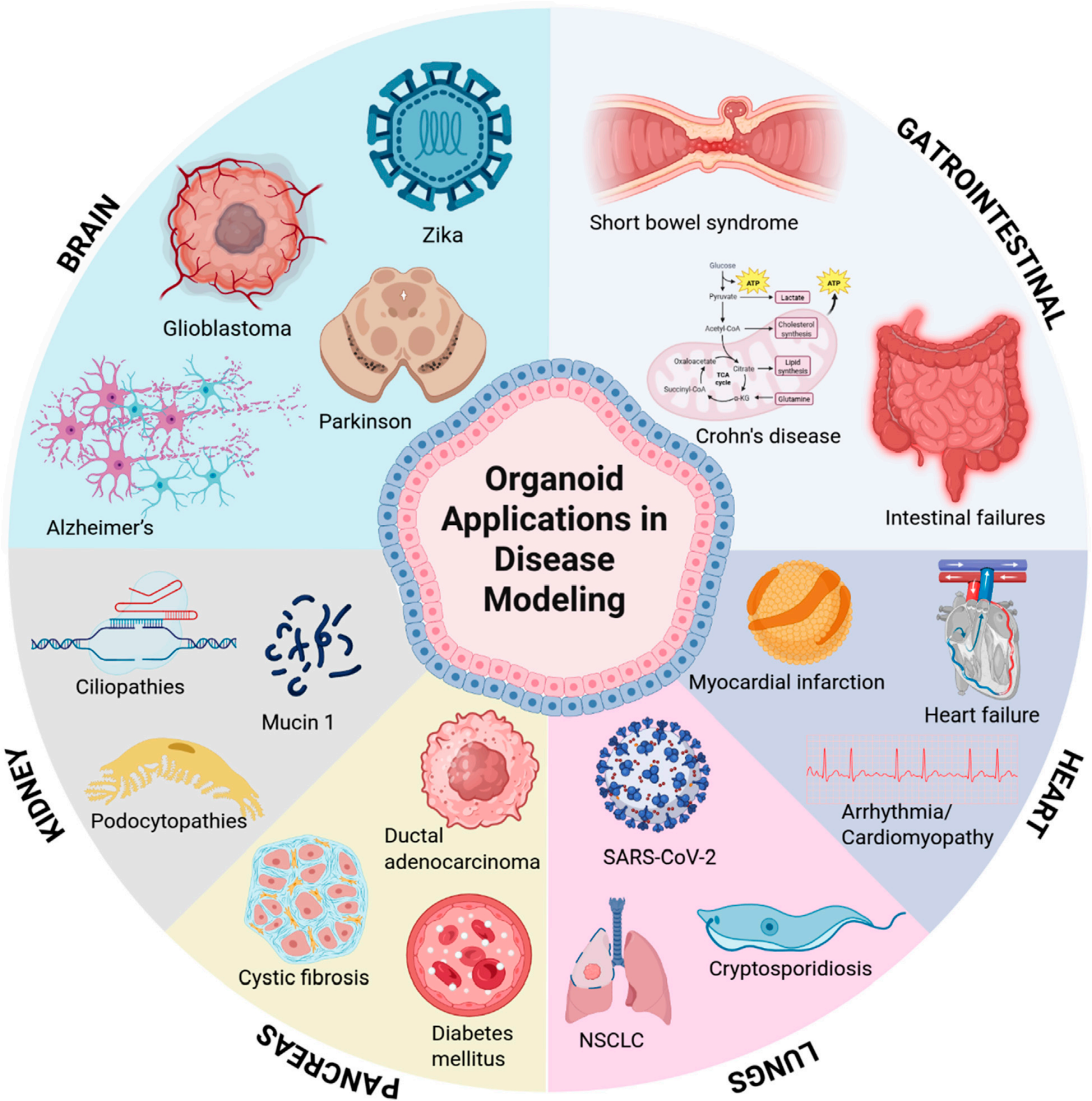
Key organ systems for disease modeling using organoid technology. Schematic representation of major organoid platforms advancing translational research, including brain, gastrointestinal tract, kidneys, pancreas, lungs, and heart.

Recent advances in organoid technology have revolutionized disease modeling by enabling the study of complex human pathologies in physiologically relevant 3D systems. Figure 2 highlights key models across major organ systems, each offering unique insights into disease mechanisms and therapeutic development. Brain organoids can model brain tumor formation and recapitulate neurodevelopmental and neurodegenerative processes such as autism and Parkinson’s disease (Bian et al., 2018; Eichmüller and Knoblich, 2022; Reumann et al., 2023). Gastrointestinal models have illuminated host-microbiome interactions and epithelial barrier dysfunctions in inflammatory bowel disease and colorectal cancer (Günther et al., 2022; Lorenzo-Martín et al., 2024a; Lorenzo-Martín et al., 2024b). Kidney organoids provide platforms for studying polycystic kidney disease and drug-induced nephrotoxicity, leveraging CRISPR-edited iPSCs to model genetic mutations (Romero-Guevara et al., 2020). Pancreatic cancer organoids capture tumor heterogeneity and metastatic potential, enabling studies on epigenetic reprogramming and stromal crosstalk (Chen et al., 2024). Lung organoids model respiratory infections and fibrotic remodeling, while cardiovascular systems replicate structural defects and cardiomyopathies, offering insights into cell-cell communication and drug screening (Vaupel et al., 2021; Pham et al., 2018). These organ-specific models exemplify the transformative potential of organoid technology in bridging molecular mechanisms with tissue-level pathophysiology.

The potential of organoids in regenerative medicine is vast, offering the first step towards bioengineered organs. Organoids can develop into macroscopic structures visible to the eye and, with proper maintenance, can continue to grow. However, one of the key challenges in organoid research is vascularization-the formation of blood vessels within organoids. Without a vascular network, organoids growing past a certain size will develop a necrotic core due to limited nutrient and oxygen diffusion. This maximum size varies depending on the metabolic needs of the particular organoid, but it is generally less than 200 μm in radius (Vilinski-Mazur et al., 2025; Vaupel et al., 2021).

To address these challenges, researchers have implemented various techniques for vascularization. One method uses an “outside in” approach by embedding an already-formed organoid in a hydrogel scaffold that has been seeded with endothelial cells. This allows vessels to grow into the organoid, as seen in Pham et al.'s cerebral organoids, which utilized iPSC-derived endothelial cells seeded in matrigel to reflect the process of vascularization in the fetal brain (Pham et al., 2018). Another approach is to co-culture cell types of interest with endothelial cells which then self-assemble into vessels. Tekebe et al. successfully developed vascularized, functional human liver buds by mixing iPSC-derived hepatic endoderm cells with human umbilical venous endothelial cells and mesenchymal stromal cells (Takebe et al., 2013). Recent research has pushed this concept by directly differentiating endothelial cells alongside the primary organoid cell type, reducing the need for multiple cell sources when designing organoids. Cakir et al. found that over-expressing endothelial transcription factor *ETV2* in human embryonic stem cells was sufficient to form vascular-like structures in brain organoids independent of media (Cakir et al., 2019). Skylar-Scott et al. streamlined this process by simultaneously differentiating neural stem cells and vascular endothelial cells from iPSCs using a method known as orthogonally induced differentiation. Here, iPSCs were pre-programmed to express either *ETV2* or neuronal transcription factor *NGN1* in the presence of doxycycline (Skylar-Scott et al., 2022). However, gene overexpression is not the only method to induce the formation of vascular networks. Homan et al. found that culturing kidney organoids under high fluid flow encouraged the proliferation of endogenous KDR^+^ endothelial progenitor cells and the formation of vascular networks, while organoids without this environmental cue failed to form vessels (Homan et al., 2019).

Beyond vascularization, organoids hold immense promise for personalized cell therapy and tissue repair. By incorporating bioengineering scaffolds, organoids have an even greater potential for creating complex *in vitro* tissue structures as an alternative to *in vivo* studies while still accurately modeling the complex nature of the body (Nwokoye and Abilez, 2024; Yin et al., 2016; Li et al., 2024).

Despite their potential, organoid research faces several limitations. One major hurdle is the scalability and reproducibility - generating consistent organoids remains a challenge due to variability in differentiation efficiency and culture conditions. Additionally, the timeline for organoid development is lengthy. While the initial generation of organoids takes approximately a week, maturation can require at least a month, depending on the cell type (Porciúncula et al., 2021). This extended culture period presents logistical challenges for high-throughput applications and clinical translation.

### 3.2 Personalized medicine

Personalized medicine tailors medical treatments to the patient, incorporating their genetic profile, lifestyle, and environment. This field leverages advancements in genomics, proteomics, pharmacogenomics, and AI-driven diagnostics to optimize treatment efficacy and minimize side effects (Arjmand et al., 2017; Akhondzadeh, 2014; Quazi, 2022; Molla and Bitew, 2024). While regenerative medicine is often applied in areas like organ regeneration, wound healing, and stem cell-based therapies for injuries or diseases (Murphy and Atala, 2014; Song and Ott, 2011; Duscher et al., 2015), personalized medicine is widely used in targeted cancer therapies, precision drug dosing, and managing chronic conditions based on individual risk factors (Besnier et al., 2021; Melero-Martin, 2022).

### 3.3 Tissue engineering: constructing functional Whole organs

Tissue engineering is an evolving field that aims to create functional tissues and organs to address critical needs such as wound healing and organ transplantation. By integrating biomaterials, stem cells, and bioengineering techniques, researchers are attempting to address these shortages. One example is the successful development of artificial skin that aimed to treat burn victims and patients with chronic skin wounds. Elaine Fuchs’ research on epidermal stem cells has provided critical insights into skin regeneration, which has informed the development of bioengineered skin substitutes (Baker, 2009; Gonzales and Fuchs, 2017; Gonzales et al., 2021; Hsu and Fuchs, 2022; Liu et al., 2023b; Yang et al., 2023; Tierney et al., 2024). The understanding of epidermal stem cells, their role in skin regeneration, along with the integration of a bioengineered scaffold, has led to the commercialization of artificial skins such as *Integra*® and *Apligraf*® (Gonzalez and Yuen, 2020; Dinh and Veves, 2006).

While bioengineered skin has successfully reached the clinical market, researchers face persistent challenges when attempting to construct larger, more complex organs such as the heart or kidney. Whole organs consist of multicellular, vascularized tissue arranged in specific patterns. While multi-cell patterning and vascularization have been achieved in organoids (see Section 3.1.2), large-scale patterning remains difficult.

One avenue to achieve complex cellular patterning *in vitro* is 3D bioprinting, which can be used to construct living tissues using bioinks composed of cells, hydrogels, and other biomaterials (Murphy and Atala, 2014). Recent advancements in 3D printing has led to printing of personalized cardiac patches by Noor et al. These patches are personalized using patient derived ECMs and are embedded with iPSCs (Noor et al., 2019). However, 3D printing has limitations. For one, printing a complex and living tissue is complicated and cannot accurately reflect the microenvironment of cells within the body. Additionally, challenges such as biocompatibility and biomaterial stability remain as obstacles to printing fully functional tissues. Despite these limitations, Lee et al. built a complex collagen scaffold that replicated the components of a human heart using a freeform reversible embedding of suspended hydrogels (FRESH) (Lee et al., 2019). These FRESH 3D-bioprinted hearts replicated patient-specific heart anatomies from capillaries to the full organ itself (Lee et al., 2019).

While tissue engineering has made significant improvements in developing functional tissues, including artificial skin and vascularized organoids, the goal of creating fully functional, transplantable organs remains a challenge. The integration of co-culturing methods, bioengineers, scaffolds, and vascularization techniques has laid groundwork for further advancements. Additionally, the emergence of 3D bioprinting as a tool for fabricating patient-specific tissues has opened new possibilities for personalized regenerative medicine.

### 3.4 Clinical translation of stem Cell therapies: emerging trials and real-world applications

Recent clinical trials demonstrate the rapid progress in translating stem cell technologies into therapeutic applications, addressing critical challenges in regenerative medicine. High-profile trials in retinal repair, pancreatic islet transplantation, and cardiac regeneration illustrate how stem cell research is moving toward real-world clinical use. One of the most recent advances is BlueRock Therapeutics and its investigational stem cell therapy Bemdaneprocel for Parkinson’s disease. The treatment consists of implanting ESC-derived dopamine-producing neurons directly into the brains of patients. Their positive Phase I results show treatment tolerability, implanted cell survival, and motor function improvements. The therapy is now progressing to Phase III trials to assess efficacy and safety in a sham surgery-controlled study involving 102 patients with moderate Parkinson’s disease (Quazi, 2022; Molla and Bitew, 2024).

A successful example of stem cell therapy for retinal diseases is the Phase I trial conducted by UC Davis Health. They demonstrated that autologous CD34^+^ stem cells isolated from bone marrow can be safely injected into the eyes of patients with retinitis pigmentosa. Four out of seven participants showed measurable improvements in vision, confirming safety and potential therapeutic benefits (News, 2024). Similarly, BlueRock Therapeutics LP, in collaboration with Opsis Therapeutics and FUJIFILM Cellular Dynamics, is conducting clinical trials on iPSC-derived photoreceptor therapies for retinal diseases, including retinitis pigmentosa and cone-rod dystrophy (Ophthalmology and Visual Sciences, 2024). Luxa Biotechnology LLC is also advancing its RPESC-RPE-4W retinal pigment epithelial stem cell therapy for dry age-related macular degeneration (AMD). Early results from Luxa’s Phase 1/2a trial demonstrated significant vision improvements in patients with severe AMD, leading to the FDA granting the therapy Regenerative Medicine Advanced Therapy (RMAT) designation (Business Wire, 2025). In contrast, a separate trial funded by the Highway Program for Realization of Regenerative Medicine investigated iPSC-derived retinal pigment epithelial (RPE) cell sheets in a patient with neovascular AMD. At 1 year post-transplantation, imaging confirmed the sheet remained intact. However, the patient’s best-corrected visual acuity showed no change, and cystoid macular edema persisted (Mandai et al., 2017). This highlights key differences in therapeutic approaches and outcomes between the cell suspension therapies and iPSC-derived sheet transplants for AMD subtypes.

An innovative example of progress in pancreatic islet transplantation comes from several clinical trials exploring advanced approaches to treat type 1 diabetes (T1D). The Edmonton Protocol remains foundational, achieving glycemic control and insulin independence in many patients through hepatic portal vein transplantation of donor islets (Cayabyab et al., 2021). Vertex Pharmaceuticals is advancing stem cell-derived islets encapsulated in immune-protective devices, such as PEC-Encap™ and PEC-Direct™, which eliminate the need for immunosuppression while addressing challenges like fibrosis (Goetz and Schork, 2018; Commissioner O, 2024). In preclinical studies, Weill Cornell Medicine demonstrated that adding reprogrammed vascular endothelial cells (R-VECs) to subcutaneous islet transplants significantly improved graft survival and reversed diabetes in mice, laying the groundwork for safer and more durable transplantation methods (WCM Newsroom, 2025). These advancements highlight the potential of pancreatic islet transplantation to improve glycemic control and reduce insulin dependence, though challenges such as limited donor availability and immune rejection persist (Wang et al., 2024a).

In a landmark clinical translation of stem cell therapies, researchers successfully generated patient-derived islets using chemically induced pluripotent stem cells and transplanted them into an abdominal site, achieving functional engraftment in a participant with diabetes. This approach restored exogenous insulin-independent glycemic control, with the patient maintaining stable blood glucose levels and meeting all pre-defined safety and efficacy endpoints at the 1-year follow-up (Wang et al., 2024b). This work highlights the potential of autologous stem cell-derived islet transplantation to provide durable insulin independence while addressing key challenges in cell manufacturing and immune compatibility.

Cardiac regeneration has also seen significant advancements, with preclinical and early-phase trials of iPSC-derived cardiac patches demonstrating graft survival and functional integration in patients with end-stage heart failure (Miyagawa et al., 2022; Tempesta, 2025; European Medical Journal, 2025). These advanced cardiac patches are engineered with conductive materials, such as graphene oxide-modified scaffolds, and rely on promoting maturation and uniforming the conduction of cardiomyocytes to reduce post-transplant arrhythmias (Bois et al., 2025).

Patients have been receiving hPSC-derived products since 2010, with 83 products undergoing testing in 115 clinical trials worldwide as of December 2024. Over 1,200 patients with 34 different conditions have been treated, receiving a cumulative dose of at least 190 billion cells and 200 billion platelets. These therapies have generally been safe and well-tolerated, even with long-term follow-up. The therapeutic landscape is expanding beyond Central Nervous System (CNS) and ocular applications to include immune, cardiac, and endocrine cell therapies, with promising efficacy data emerging for conditions like diabetes, epilepsy, Parkinson’s disease, and Age-Related Macular Degeneration (AMD). Challenges remain in designing ethical phase III trials and scaling manufacturing processes while ensuring sustainable pricing models (Kirkeby et al., 2025).

Many biotechnology companies are extensively researching cell-based therapies, positioning them as transformative alternatives to traditional small-molecule treatments due to their ability to target complex biological systems with higher specificity and adaptability (Quazi, 2022; Molla and Bitew, 2024). This shows how the field is moving toward targeted approaches that focus on what cells need to thrive, such as avoiding immune rejection, getting proper blood supply, and maturing into functional tissue. By tackling these challenges, researchers are bringing us closer to translating lab breakthroughs into life-changing clinical treatments.

#### 3.4.1 Emerging solutions: biomaterial-assisted delivery

Stem cell delivery strategies currently rely on two primary approaches: intravenous (i.v.) infusion and direct local administration into target organs. Systemic i. v. delivery offers non-invasive access to widespread tissues but suffers from poor cell survival due to immune clearance, entrapment in off-target organs (e.g., lungs), and limited retention at injury sites (Huerta et al., 2023; Bagno et al., 2022; Leibacher and Henschler, 2016). Local administration (e.g., intra-articular, intramyocardial) improves site-specific engraftment but risks invasive procedural complications, cell leakage, and uneven distribution within damaged tissues (Li et al., 2021a; Terrovitis et al., 2010; Ashammakhi et al., 2019). Both methods face persistent challenges in maintaining therapeutic cell populations and preventing off-target effects, which undermine clinical efficacy.

Emerging backpack technology addresses these limitations by engineering cells with surface-conjugated biomaterials that enhance survival and precision. Backpacks can also incorporate targeting ligands (e.g., peptides binding to upregulated integrins in ischemic tissues) to improve site-specific retention, reducing off-target migration (Li et al., 2021b; Anselmo et al., 2015). By synergizing the scalability of i. v. delivery with the precision of localized administration, backpack-modified stem cells offer a transformative strategy to bridge the gap between preclinical promise and clinical reality.

##### 3.4.1.1 Backpack molecules for precision delivery

One interesting approach for targeted drug delivery is cell-mediated therapy, where the surface of living cells are engineered with ligands of interest (e.g., nanoparticles) to improve therapeutic potency (Polak et al., 2015). This engineering approach has been inspired by the mammalian pathogens hemotrophic mycoplasmas, which bind to the erythrocyte surface and can remain in circulation for several weeks. (Chambers and Mitragotri, 2004). The Backpack molecules, also known as “Cellular backpacks”, have shown promising applications especially in treating autoimmune diseases and enhancing tissue repair.

Backpack molecules, pioneered by Dr. Samir Mitragotri, are disc-shaped microparticles engineered to adhere to immune cell surfaces (Kenry et al., 2022; Klyachko et al., 2017). These structures are typically comprised of multiple layers of carefully selected polymers, such as poly (lactic-co-glycolic acid) (PLGA) and polyvinyl alcohol (PVA), with a specialized cell-adhesive layer (Kapate et al., 2023). The precise control of size and shape is critical in backpack design, as these parameters must be optimized to effectively trigger cellular responses without impeding normal cell function. This delicate balance allows backpacks to modulate immune cell behavior while maintaining the cell’s ability to navigate through tissues and perform its intended functions (Kapate et al., 2023; Kenry et al., 2022; Brenner et al., 2018).

**FIGURE 3:**
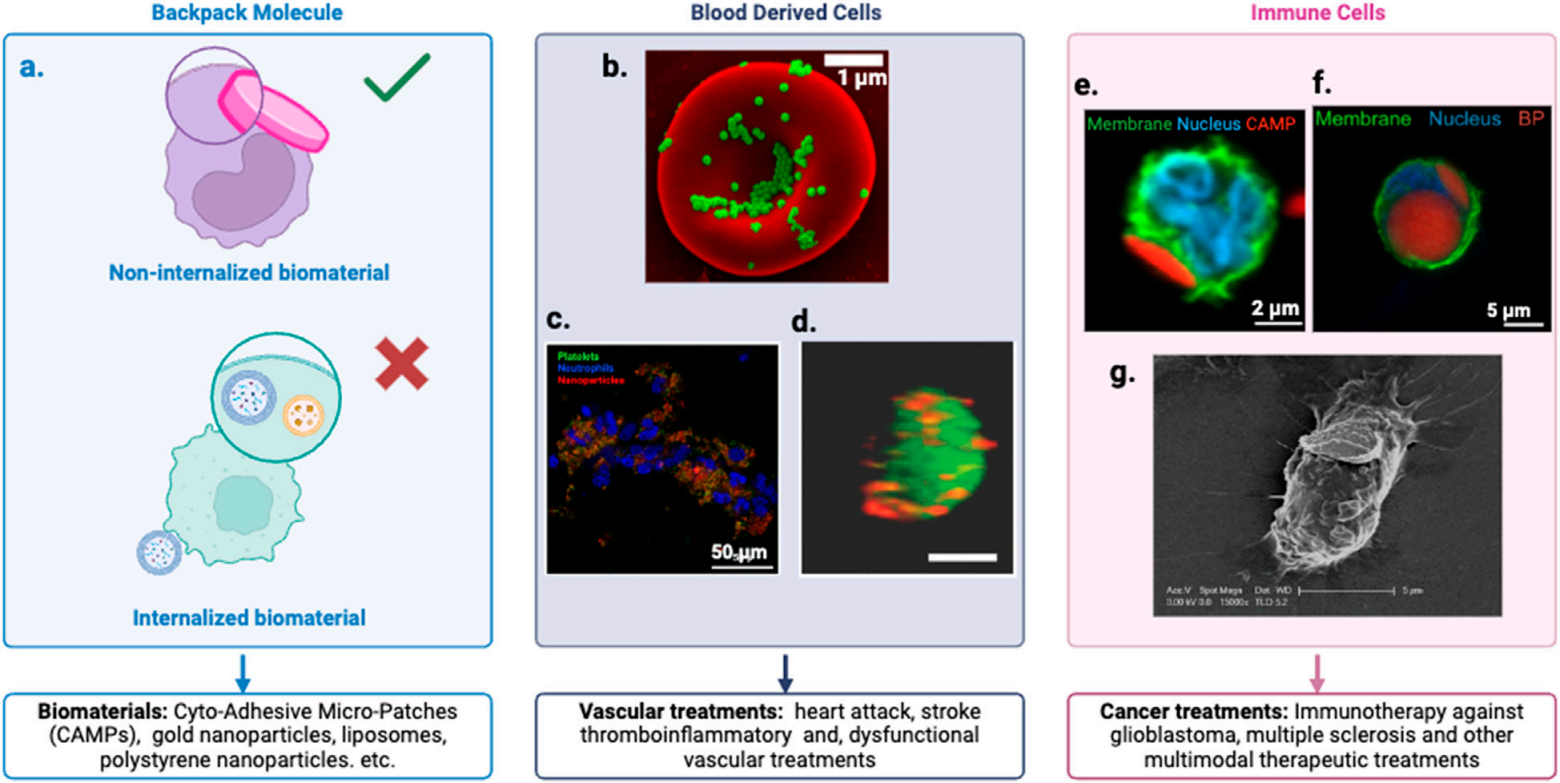
Cellular Backpack Technology. **(a)** Customizable cell lines and backpacks with specific ligands and therapeutic payloads tailored to target cells and applications. Defined as backpack molecules when the biomaterial remains external to the cell, not internalized. **(b)** Scanning electron micrographs of PS-NPs and nanogels attached to the surface of murine RBCs (Brenner et al., 2018). **(c)** Fluorescence images of platelet-inspired nanoparticles targeted to thrombo inflammatory (e.g., binding platelets and neutrophils) pathologies (Raghunathan et al., 2022). **(d)** A confocal photograph demonstrating a stable conjugation of Dil-labeled multilamellar lipid nanoparticles (red) conjugated onto the surface of a CFSE-labeled ECFC (green) (Bui et al., 2022). **(e)** Confocal image of a neutrophils (NEs) after incubation with Cyto-Adhesive Micro-Patches (CAMPs). The right image indicates representative NE with an attached CAMP (Fukuta et al., 2025). **(f)** Confocal micrograph of monocyte (membrane: green, nucleus: blue) with backpack (red) (Kapate et al., 2023). **(g)** Illustration of an immune cell carrying a nanoparticle ‘backpack’ (purple) deep into tissues to target specific sites of injury and disease. Credit: Wyss Institute at Harvard University (Doshi et al., 2011).

The defining characteristic of a Backpack molecule lies in its unique attachment mechanism to cells, but equally important is the synergistic relationship between the cell and the biomaterial. This cell-biomaterial complex functions as a unified treatment modality, where the components complement each other’s strengths. In some applications, the cell serves as a “stealth” carrier, effectively hiding the biomaterial and enhancing its biocompatibility (Figure 3), thereby improving circulation time and reducing immune recognition (Raghunathan et al., 2022; Fukuta et al., 2025) Alternatively, when the cell itself is the primary therapeutic agent, the attached biomaterial acts as a guide, leveraging its targeting features to direct the cell to specific tissues or disease sites (Caplan, 2017; Kapate et al., 2023). This symbiotic arrangement allows for more precise and effective treatments, combining the biological functions of cells with the engineered properties of biomaterials to create a versatile and potent therapeutic platform.

##### 3.4.1.2 Targeted backpack molecules

Backpack molecules adhere to the cell surface without being internalized, enabling prolonged effects and sustained interactions. To enhance cell-specific adhesion, backpack molecules can be functionalized with a variety of targeting reagents, including antibodies (e.g., anti-CD45), peptides, aptamers, and small molecules [Refer to Table 1 **]**. This versatile functionalization allows researchers to tailor backpack molecules for specific cell types or targets, significantly improving their precision and efficacy in various biomedical applications. The non-internalized attachment and the ability to incorporate diverse targeting moieties make backpack molecules a powerful tool in drug delivery (Anselmo et al., 2015), cell therapy, and tissue engineering (Shields et al., 2020), offering new possibilities for targeted interventions at the cellular level (Kapate et al., 2023). As discussed above, the versatility of backpack molecules has led to their application in various biomedical fields, each leveraging the unique cell-surface attachment mechanism to address specific therapeutic challenges. These applications can be broadly categorized into four main areas: targeting immune diseases, enhancing drug delivery, modulating immune responses, and promoting tissue repair.

**TABLE 1:** Backpack molecules, initially designed for immune cells, have expanded to include stem cells for targeted drug delivery. These structures attach to cell membranes, enhancing their therapeutic potential by leveraging their natural homing abilities.

Cell type	Backpack	Application	Ref
Macrophages (RAW 264.7)	Polymer patches	Penetration of Brain Blood Barrier (BBB)	Klyachko et al. (2017)
Autologous macrophages (MΦs)	Drug crystals, bacteria, gold particles, inert emulsions or liposomes	Drug delivery	Lee et al. (2016)
Porcine bone-marrow-derived macrophage (BMDMs)	PLGA/PVA	Traumatic brain injury (TBI)	Kapate et al. (2024)
Bone marrow–derived macrophages (BMDMs)	PLGA/PVA/PLGA/HA-Ald/PAH	Immunotherapy/Cancer treatments	Shields et al. (2020)
Monocytes (WEHI-265.1)	(PMAA2/PVPON2)30.5 (PAH3/MNP4)10.5 (PAA4/PAH-biotin4)8	Alzheimer’s disease, Parkinson’s disease, and arthritis	Anselmo et al. (2015)
Primary murine bone-marrow-derived monocytes (BMMs)	PLGA–PEG–Mal	Antitumor therapy	Kapate et al. (2025)
Differentiated Monocytes from Bone marrow (mice)	PLGA/PVA	Multiple sclerosis (MS)	Kapate et al. (2023)
Macrophages (BMDMs)	SPIONs/PLGA	Targeting, Imaging, and Immunotherapy	Day et al. (2024)
Blood-borne macrophages	Phospholipidids, Indinavir (IDV) nanoparticles	HIV-associated neurocognitive disorders, Penetration of BBB	Dou et al. (2009)
Mouse peritoneal inflammatory macrophages	Liposomal doxorubicin (LP-Dox)	Cancer therapy	Jinhyang et al. (2012)
Monocytes from Buffy Coat	Gold-silica nanoshells	Brain metastases, Penetration of BBB	Choi et al. (2012)
Red blood cells (RBCs)	Polystyrene (PS) nanoparticles	Blood pharmacokinetics and vascular delivery of nanoparticles, Lung targeting	Anselmo et al. (2013)
Red Blood Cells (erythrocytes)	Polystyrene (PS) nanoparticles	Avoid reticuloendothelial system (RES) clearance	Chambers and Mitragotri (2004)
Marrow-isolated adult multilineage inducible (MIAMI) cells	Poly-lactic acid NPs (PLA-NPs) and lipid nanocapsules (LNCs)	Glioma therapy, brain tumors	Roger et al. (2010)
T-cells	Liposomes and liposome-like synthetic nanoparticles	Tumor eradication	Stephan et al. (2010)
T-cells	NSC-87877 loaded liposomes	Prostate cancer	Stephan et al. (2012)
Neutrophils (NEs)	CAMPs composed of PLGA and PLGA-PEG-maleimide	Immunotherapy against glioblastoma	Fukuta et al. (2025)
Endothelial Colony-Forming Cells (ECFCs)	Liposomal Nanoparticles (LNPs)	Rejuvenate circulating vascular progenitor cells	Bui et al. (2022)

So far, endothelial progenitor stem cells have been used with this technology (Bui et al., 2022), but the potential integration of stem cells could open up new possibilities for treating various diseases, offering a promising frontier in therapeutic applications.

##### 3.4.1.3 Integration with clinical-Grade biomaterials

When producing backpack molecules, there are multiple strategies that can be used to incorporate nanomaterials onto the cell surface. These include: adsorption onto cell membrane, internalization, maleimide–thiol covalent coupling, ligand-receptor interactions, covalent coupling, and internalization. The simplest method to create a backpack molecule is adsorption, since it involves a passive interaction between the nanomaterial and the surface of the cell. This method relies on electromagnetic interactions, which are usually mediated via hydrophobic interactions, van Der Waals forces, and hydrogen bonding (Takeuchi et al., 2021; Bell et al., 2023).

Another method that doesn’t require extensive cell modification is by using ligand-receptor interactions. This strategy is advantageous for scaled-up production, as altering the attachment ligand allows the same molecules to attach to multiple cell types. On the other hand, caution must be used when identifying potential receptors. If the ligand is targeted to a widely-produced receptor, it can lead to the undesired accumulation of cells in other organs and promote teratoma formation in the case of stem cells due to their pluripotency (Meyer-Hermann, 2018).

Covalent coupling involves the modification of both particle and cell to promote a stronger binding than either adsorption or ligand-induced binding. One of the most popular examples of this is by using thiol-reactive maleimide groups on the nanomaterial. This mechanism takes advantage of the presence of the thiol groups on the membrane surface of certain cell types (ej. T-Cells, RBS) to form a covalent bond. This thiol-mediated covalent bonding shows prolonged surface retention and avoided particle internalization (Caplan, 2017; Dou et al., 2009). Additionally, the stronger binding provided by covalent bonding limits the detachment and uptake of particles in non-target tissues.

Another technique that has been used to produce Backpack molecules is the internalization of nanoparticles or “Trojan Horse” method (Choi et al., 2012). This technique uses the phagocytic nature of a cell to engulf a foreign nanomaterial. This method leaves the cell membrane unaltered and can potentially protect the nanoparticles from interacting with non-target tissue *in-vivo* (Anselmo and Mitragotri, 2014).

##### 3.4.1.4 Challenges and future directions

The application of backpack technology in stem cell engineering faces several key challenges. Foremost is the need to create tailored backpack designs for different stem cell types without compromising their phenotype or essential characteristics. Ensuring the backpacks remain attached during cell differentiation and proliferation is equally crucial. Additionally, developing efficient, scalable production methods for clinical applications remains a significant hurdle.

Looking ahead, the future of backpack technology in stem cell engineering holds exciting possibilities. A primary focus is the development of patient-specific backpack-stem cell combinations, with backpack payloads customized based on individual genetic profiles (Liu et al., 2024a). This approach could significantly advance personalized medicine. Future research could explore potential synergies between backpack-modified stem cells and other therapeutic modalities, such as small molecules or growth factors, which might enhance treatment efficacy (Caplan, 2017; Oduk et al., 2018). Furthermore, the integration of imaging agents into backpacks for real-time cell tracking could revolutionize our ability to monitor stem cell therapies *in vivo* (Gamage et al., 2021), providing crucial insights into cell behavior and treatment outcomes.

## 4 Advances in stem cell culture and engineering

### 4.1 Biomaterials

A material designed to interact with biological systems, to evaluate, treat, or even substitute any organ or tissue in a living organism is considered a biomaterial. These types of materials have various applications in medicine and biomedicine (Meyer et al., 2019; Deng, 2017). In cell culture, hydrogel scaffolds are typically utilized as biomaterials and are designed to engage with biological organisms. As such, they are subject to certain standards: (i) Biocompatibility—the material’s ability to minimize immunological rejection and function harmoniously with the host; (ii) Adequate durability related to its intended function; (iii) Bioreabsorption capacity—the ability for the body to metabolize the material; (iv) Biodegradability, which refers to its potential for biological degradation; (v) Mechanical properties that are suitable for the stresses and deformations it may encounter (Hernandez et al., 2018).

Hydrogels can be made of synthetic and natural materials (Zucker and Prendergast, 2020). In stem cell engineering, natural biomaterials are typically preferred for scaffold-cell interactions due to their specific molecular domains and architecture. Natural biomaterials can be either protein-based or polysaccharide-based. Protein-based biomaterials include bioactive molecules that replicate the extracellular environment, such as collagen, fibrin, gelatin, and keratin (Datta et al., 2020). Polysaccharide-based biomaterials are primarily derived from various sources: algae (e.g., alginate), animals (e.g., chitosan, hyaluronic acid), and other natural sources (Benalaya et al., 2024). For simplicity, we refer to “natural” biomaterials as minimally modified biological materials, while “synthetic” includes both artificial and significantly engineered naturally-sourced materials. Examples of natural biomaterials include collagen, fibrin, laminin, and alginate. Synthetic or engineered biomaterials include PEG-based hydrogels, PLLA, PLA, PLGA, PCL, and PVA. Some commercial products like Matrigel and Geltrex may contain both natural and synthetic components.

#### 4.1.1 Influence of biomaterials on stem Cell maintenance and differentiation

Biomaterials influence cell behavior by providing mechanical, chemical, biological, and other environmental cues. In the case of stem cells, biomaterials can help maintain an undifferentiated state or push cells towards a specific lineage. One way biomaterials can assist in maintaining stem cell pluripotency *in vitro* is by mimicking the stem cell niche. For example, culturing human amniotic fluid-derived stem cells (hAFSCs) on soft hydrogels causes them to express higher levels of pluripotency markers (Wang et al., 2015), while growing MSCs on micro-patterned fibronectin restricts cell spreading and prevents spontaneous differentiation (Zhang and Kilian, 2013). With this technology, biomaterials can be used to standardize the secretome of MSCs for use in tissue engineering or for clinical applications [see (Wechsler et al., 2021) for review]. Recent advances highlight topography-guided engineering as a critical regulator: Nguyen et al. demonstrated that mesoscopic collagen architectures (e.g., islands vs. fibrillar networks) direct MSC fate by modulating cytoskeletal tension and YAP/TAZ signaling (Nguyen et al., 2023). Similarly, Xu et al. showed that substrates mimicking blastocyst geometry revert primed iPSCs to a naïve state, enhancing their differentiation plasticity through geometric activation of KLF4 and TFAP2C (Xu et al., 2024).

In cases where differentiation is desired, environmental and mechanical cues provided by biomaterials can encourage stem cells towards specific lineages. For instance, hypoxia-mimicking hydrogels encourage MSCs to differentiate into chondrocytes (Sathy et al., 2019). Culturing iPSCs in soft (0.one to one kPa) 3D hydrogels mimicking pancreatic stiffness enhances pancreatic progenitor differentiation and glucose-responsive insulin secretion, while stiffer matrices bias cells toward non-endodermal fates (Anjum et al., 2016; Karbassi et al., 2020). Growth factors can also be incorporated to differentiate stem cells, as shown by the osteogenic differentiation of MSCs when grown in a bone morphogenetic protein-2 releasing scaffold (Anjum et al., 2016).

Biomaterials are also useful for maturing iPSC-differentiated cells. Maturation is a critical step for using iPSC-derived cells in tissue engineering. It not only causes them to be closer in morphology and function to native cells, but using matured cells also lowers the risk of off-target tissue formation caused by incomplete differentiation. Maturity is especially important for iPSC-derived cardiomyocytes, as these cells must be able to beat and respond to electrical signaling to match native heart tissues (Karbassi et al., 2020). To this end, several groups have utilized biomaterials to mature iPSC-cardiomyocytes (iPSC-CMs). Asaro et al. developed an electroconductive collagen-MXene (Ti_3_C_2_T_x_) material that, when combined with an external electric field, resulted in iPSC-CMs with elongated cell morphologies and increased the expression of cx43 (Asaro et al., 2023). For synthetic biomaterials, Chun et al. created a synthetic polymer matrix from different ratios of poly-ε-caprolactone (PCL), polyethylene glycol (PEG), and carboxylated PCL (cPCL). Their iPSC-CMs showed increased contractile ability (Chun et al., 2015). Recently, Iwón et al. used PCL and polyurethane nanofibrous mats to mature iPSC-CMs. Compared to traditional culture on polystyrene plates, the nanofibrous mats caused the iPSC-CMs to have increased CM morphology, gene, and protein expression (Iwoń et al., 2024).

To have the desired impact on cell differentiation and maturation, the biomaterial composition, functionalization, and structure must all be carefully designed. The next sections review commonly-used biomaterials in stem cell engineering, as well as tissue engineering strategies for building complex structures.

#### 4.1.2 Extracellular matrix (ECM)

The extracellular matrix (ECM) is a crucial component of the stem cell niche, as it can directly or indirectly influence the maintenance, proliferation, self-renewal, and differentiation of stem cells. Various ECM molecules serve regulatory functions for different types of stem cells, and the ECM’s molecular composition can be precisely adjusted to create the most suitable niche for stem cells across various tissues. Engineered biomaterials that replicate the *in vivo* characteristics of the stem cell niche offer valuable *in vitro* tools for exploring the diverse roles played by the ECM and its molecular components in regulating stem cell behavior (Gattazzo et al., 2014).

Significant advancements have been made in vascular tissue engineering and regenerative medicine over the past few decades, particularly with the development of biomaterials derived from ECM proteins. These biomaterials offer mechanical support and biochemical signals that influence vascular cell attachment, phenotype, and behavior. Initially, ECM-derived biomaterials were utilized as two-dimensional (2D) coatings to enhance cell adhesion on tissue culture polystyrene dishes. Subsequent developments have resulted in three-dimensional (3D) ECM-derived biomaterials that exhibit enhanced tunability, allowing them to more accurately mimic the dynamics, composition, and structure of native ECM (Wu et al., 2021).

##### 4.1.2.1 2D and 3D ECMs

There are benefits to using a 2D hydrogel coating over a 2D plate culture. For example, their enhanced cell-cell interaction which allows them for more biologically relevant cellular behavior and communication (Caliari and Burdick, 2016). Additionally, hydrogels coatings are able to replicate the physical and biochemical properties of the ECM providing a more physiologically relevant environment for cells, which can influence their attachment, proliferation, and differentiation (Scheidecker et al., 2024; Johnson, 2008). Most common culture methods for stem cells employ 2D techniques using plastic, which fails to replicate *in vivo* environments.

In comparison, 3D cultures have a significant advantage: they more accurately mimic the complex interactions that occur between cells and between cells and their surrounding environment (matrix) as they would in a living organism. When grown in 3D cultures, stem cells display enhanced viability, differentiation potential, and more accurate tissue-like behavior compared to traditional 2D cultures (Kapałczyńska et al., 2018). These unique characteristics make 3D-cultured stem cells particularly valuable for research and potential therapeutic applications (Ylostalo, 2020). Table 2. presents a comprehensive list of commonly used ECMs, along with their sources (natural or synthetic) and typical cell lines used with each biomaterial.

**TABLE 2:** Summary of some of the most commonly used biomaterials for culturing and co-culturing stem cells across various applications, from cell differentiation to disease modeling. [Refer to glossary for standardized cell line acronyms].

Biomaterial	Dimension	Cell lines	Type of ECM	Ref.
Collagen	2D/3D	HGFs, HUVECs, HPC, MSCs	Natural	Leisten et al. (2012), Valdoz et al. (2021), Masson-Meyers et al. (2022), Turnbull et al. (2014), Hoshiba and Yamaoka (2019)
Matrigel	2D/3D	NSCs, ESCs, iPSCs	Natural/Synthetic	Lee et al. (2015), Wang et al. (2020), Saha et al. (2024)
Fibrin	2D/3D	iPSC, MSCs, ECs	Natural	Barsotti et al. (2010), Gandhi et al. (2019), Chandrababu et al. (2020)
Gelatin	2D	MSCs, NSCs, iPSCs	Natural	Lee et al. (2015), Arkenberg et al. (2022), Zhao et al. (2015)
PEG-Based Hydrogel	2.5D, 3D	hADSCs, MSCs	Synthetic	Gao et al. (2015a), Gao et al. (2015b), Hassan et al. (2013), Blache et al. (2022)
Alginate	2D, 3D	hMSCs, hiPSCs	Natural	Twizeyimana et al. (2022), Galocha-León et al. (2024), Zhu et al. (2025), Pangjantuk et al. (2024)
Chitosan	2D, 3D	hiPSCs, MSC, hNSCs, ADSCs	Natural/Synthetic	Zhu et al. (2024), Ando et al. (2023)¸ Li et al. (2013), Chang et al. (2021)
Laminin	2D	hPSCs, ESCs, hES	Natural	Rodin et al. (2014), Yap et al. (2019), Miyazaki et al. (2017)
Fibronectin	2D	LECs, ESCs, MSCs, ADSCs	Natural	Saha et al. (2023), Basoli et al. (2021), Tragoonlugkana et al. (2024), Singh and Schwarzbauer (2012)
Hyaluronic Acid Hydrogel (HA)	3D	MSCs, LECs, ECFCs	Natural	Pangjantuk et al. (2024), Alderfer et al. (2024), Hanjaya-Putra et al. (2011), Fan et al. (2023), Demirel et al. (2024)
VitroGel RGD	2D, 3D	MSC, BMSCs, PSCs	Synthetic	Kim et al. (2023), Wang et al. (2024c), Damerau et al. (2024), Fu et al. (2023)
Poly-L-Lactic Acid (PLLA)	2D, 3D	MSCs, HVSMCs	Synthetic	Capuana et al. (2022), Lee et al. (2009)
Silk Fibroin	3D	hNPs, hMSCs, HUCMSCs	Natural	Liu et al. (2022), Patil and Singh (2019), Subia et al. (2015)
Decellularized ECM	2D, 3D	NSCs, MSCs	Natural	Assunção et al. (2020), Giobbe et al. (2019), Baiguera et al. (2014), De Waele et al. (2015)
Chondroitin Sulfate (CS)	3D	MSCs, ESCs, UCB-MNCs	Natural/Synthetic	Nakanishi et al. (2022), Izumikawa et al. (2014), Wang and Yang (2017), Ai et al. (2023)
Keratin	3D	MSCs, hASCs	Natural	Ghosh et al. (2017), Bhat et al. (2024), Lin et al. (2019), Bochynska-Czyz et al. (2020)
Collagen I	2D, 3D	NIH-3T3, DPSCs, MSCs, NSCs, hESCs, iPSCs	Natural	Galocha-León et al. (2024), Liu et al. (2012), Lin et al. (2004), Roether et al. (2018), Mochizuki et al. (2020)
Decellularized Dermis	3D	HUVECs, hCPCs, ASCs, hMSC	Natural	Giavaresi et al. (2013), Doornaert et al. (2019), Belviso et al., 2020, Patel et al. (2021)
Geltrex	2D	iPSCs	Natural/Synthetic	Gandhi et al. (2019), Kogut et al. (2014)
Gelma	3D	MSCs, GSCs, iPSCs, BMSCs	Synthetic	Bupphathong et al. (2022), Shah et al. (2021), Fan et al. (2018), Zhou et al. (2020)
Polylactide (PLA)	3D	NSCs, MSCs, DPSCs	Synthetic	Salerno et al. (2016), Kim et al. (2022), Salerno et al. (2015)
Poly (lactic-co-glycolic acid) (PLGA)	2D, 3D	hASCs, DPSCs, MSCs	Synthetic	Li et al. (2017), Hess et al. (2017), Ciardulli et al. (2020)
Poly (ε-caprolactone) (PCL)	2D, 3D	hADSCs, MSCs	Synthetic	Ebrahimi et al. (2022), Qin et al. (2022), Carter et al. (2019)
Poly (vinyl) alcohol (PVA)	3D	iPSCs, MSCs, SCCs	Synthetic	Dattola et al. (2019), Asghari et al. (2022), Ziloochi Kashani et al. (2020)
Polyethylene glycol (PEG)	2D,3D	ESCs, MSCs, hPSCs	Synthetic	McKee et al. (2019), Lei and Schaffer (2013), Whitehead et al. (2018)

#### 4.1.3 3D printing in Biomaterial engineering

The extracellular matrix (ECM) plays a multifaceted role in cellular processes, influencing everything from cell adhesion to survival. In the field of tissue engineering, the application of ECM and ECM-mimicking scaffolds has led to split approaches: scaffold-based and scaffold-free methodologies. Scaffold-free techniques excel in generating consistent cell aggregates, offering significant potential for high-throughput, reproducible drug screening and disease modeling. However, the absence of ECM in these systems can hinder the survival and proliferation of certain cell types.

**FIGURE 4:**
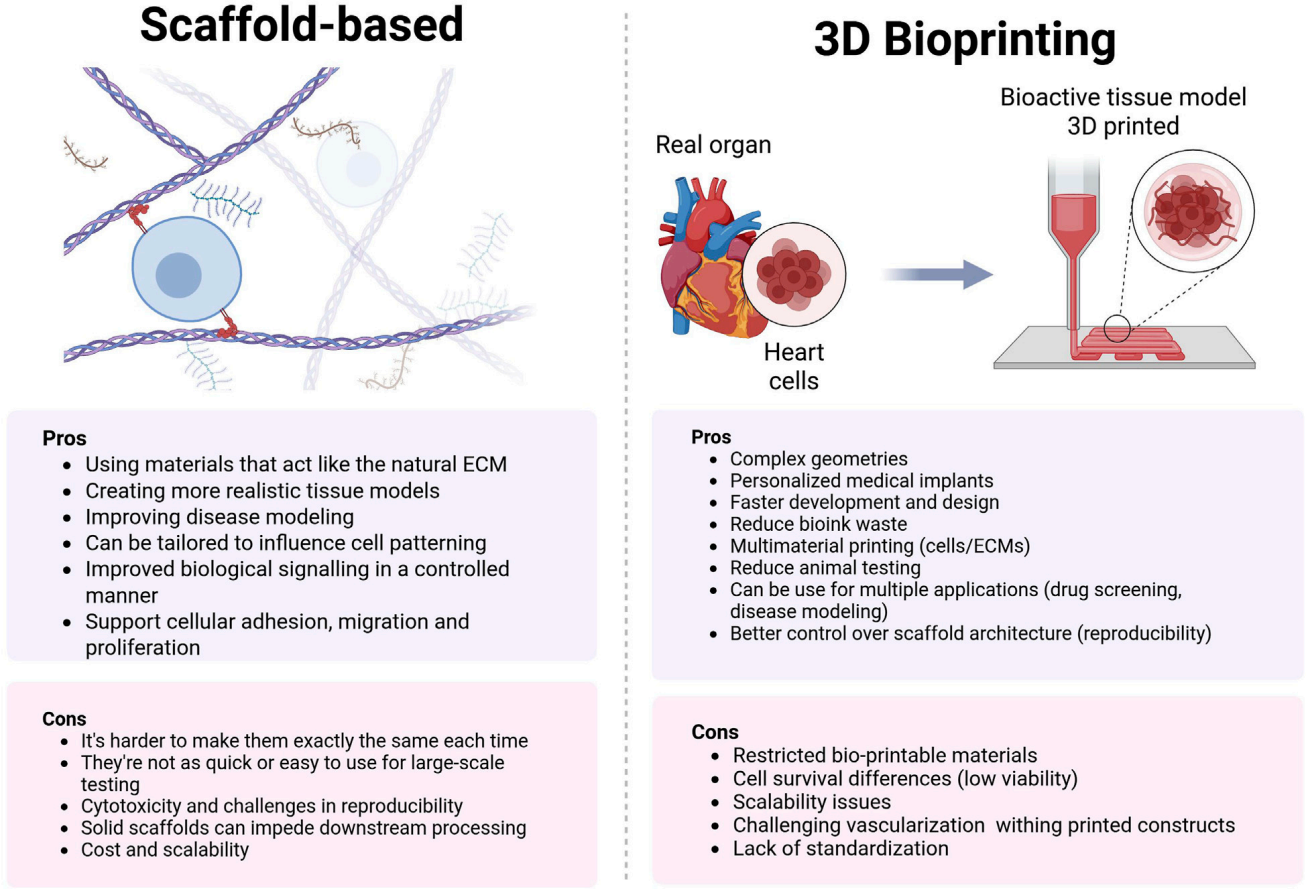
Comparison of ECM scaffolds vs. 3D bioprinting: Balancing biomimicry and precision in tissue engineering. ECM scaffolds offer natural cell environments but lack structural control, while 3D bioprinting enables complex architectures but faces material and scalability challenges.

To address this limitation, tissue engineers employ scaffolds that emulate the native ECM, resulting in organotypic models that demonstrate enhanced reliability in disease modeling. These scaffold-based approaches, while more physiologically relevant, come with trade-offs in terms of reproducibility and throughput compared to their scaffold-free counterparts. This dichotomy in tissue engineering approaches highlights the ongoing challenge of balancing physiological accuracy with experimental efficiency and reproducibility (Valdoz et al., 2021). Figure 4.

3D bioprinting combines 3D printing principles with biological materials to create living tissue structures. This invention was pioneered by Dr. Hideo Kodama in 1981, though Chuck Hull is credited with filing the first patent for 3D printing in 1984 (RAISE3D, 2024). The first bioprinted cells were human embryonic stem cells in 2013. The concept gained significant attention when 3D printers became more accessible (Faulkner-Jones et al., 2013). Tal Dvir from Tel Aviv University is a notable researcher in the field, having printed a small-scale, cellularized model of the human heart, complete with chambers and major blood vessels, in 2019 (Cellink, 2025).

Scaffold-free methods excel in generating uniform cell aggregates for high-throughput screening but often lack the mechanical and biochemical cues needed for long-term cell viability. Conversely, scaffold-based strategies leverage ECM-mimetic hydrogels to enhance physiological relevance, albeit with trade-offs in reproducibility (Valdoz et al., 2021). Recent advances in 3D bioprinting bridge this gap by enabling precise spatial patterning of stem cell-derived tissues with native-like cell densities and embedded functional architectures. For example, Skylar-Scott et al. (2019) demonstrated organ-specific bioprinting of cardiac patches with >10^8 cells/mL and perfusable vascular channels using sacrificial bioinks (Skylar-Scott et al., 2019), while their following work introduced orthogonal differentiation cues to pattern vascularized organoids within printed constructs (Skylar-Scott et al., 2022). Daly et al. further advanced this by fusing iPSC-derived spheroids in self-healing hydrogels, achieving high-density tissues with heterogeneous zonation (Daly et al., 2021).

Three major approaches to creating 3D bioprinted materials are biomimicry, autonomous self-assembly, and mini-tissue building blocks.

Biomimicry in 3D bioprinting aims to create precise replicas of cellular and extracellular components found in natural tissues or organs. This approach focuses on developing physiologically accurate biomaterials and gradients that closely mimic the native environment. To achieve success, biomimicry requires meticulous replication of biological tissues at the microscale level. This involves accurately reproducing the complex architecture of tissues, including the spatial arrangement of multiple cell types, the composition and structure of the extracellular matrix, and intricate networks of blood vessels and other supporting structures. By faithfully recreating these elements, biomimicry strives to produce functional tissue constructs that closely resemble their natural counterparts in both structure and function (Murphy and Atala, 2014; Ingber et al., 2006).

Autonomous self-assembly draws inspiration from embryonic organ development processesThis approach often employs a ‘scaffold-free’ method, utilizing self-organizing cellular spheroids. This technique relies on harnessing the innate ability of cells to organize into complex structures as occurs during embryogenesis (Steer and Nigam, 2004). Successful implementation requires a deep understanding of the developmental mechanisms governing embryonic tissue formation and organogenesis. For successful self-assembly, bioprinted constructs must carefully control cell signaling, mechanical forces, and nutrient gradients. This approach aims to produce physiologically relevant tissue structures by allowing cells to guide their own organization, leading to functional tissue constructs (Murphy and Atala, 2014; Gonzalez and Yuen, 2020).

Recent advancements in 3D bioprinting have focused on improving printing speed and cell viability. Gao et al. (2024) developed HITS-Bio (High-throughput Integrated Tissue Fabrication System for Bioprinting), a multiarray bioprinting technique that can position multiple spheroids simultaneously, achieving speeds ten times faster than existing methods while maintaining a greater than 90% cell viability (Kim et al., 2024a).

The combination of peptide self-assembly and 3D printing is an important advancement in biomedical engineering (Farsheed et al., 2023; Irukuvarjula et al., 2025; Farsheed et al., 2024). This technology improves the accuracy and efficiency of creating biocompatible structures and opens up new opportunities for developing advanced medical devices and solutions in tissue engineering.

The mini-tissues approach represents a sophisticated method for constructing complex biological structures by utilizing smaller, functional building blocks (Farsheed et al., 2023; Muir et al., 2022; Isik et al., 2023). This technique is grounded in the understanding that organs and tissues are composed of discrete, functional units, such as kidney nephrons. The primary goal is to build larger, more complex constructs through rational design, self-assembly, or a combination of both strategies. Two major approaches are employed 1) organizing many small, self-assembling cellular spheroids into one macro-tissue; 2) designing high-resolution tissue units into functional larger structures. Combinations of these strategies are likely necessary to successfully print 3D biological structures which combine functional, structural, and mechanical components (Gonzalez and Yuen, 2020; Business Wire, 2025). By leveraging the inherent organizational capabilities of cells and precise engineering techniques, the mini-tissues approach offers a promising pathway to creating more physiologically accurate and functionally complex tissue constructs.

##### 4.1.3.1 Multi-component bioinks

To achieve any of the techniques mentioned above, it’s necessary to think about the design of multi-component Bioinks. Multi-component bioinks are engineered to combine the beneficial properties of various biomaterials, enhancing the functionality and printability of 3D bioprinted constructs. They can be either natural or synthetic polymers but can be combined to create a bioink or granular hydrogels that can provide both biocompatibility and mechanical strength (Pan et al., 2021; Muir et al., 2022; Isik et al., 2023).

Another way to create a Multi-component bioinks is to use a decellularized Extracellular Matrix (dECM). dECM-based bioinks are rich in bioactive proteins and factors, reducing the risk of immune rejection and providing a suitable environment for tissue regeneration. This can be translated into promoting cell adhesion, proliferation, and differentiation, closely mimicking the native ECM (Wan et al., 2024).

To develop advanced bioinks for 3D printing complex tissues and organs it is important to incorporate bioactive molecules like growth factors, which are crucial for guiding cell behavior and promoting tissue formation, as well as control their release kinetics. Controlled release of bioactive molecules can be achieved through encapsulation in delivery vehicles such as nanoparticles, microparticles or even Small Extracellular Vesicles (sEVs) (Koons and Mikos, 2019; Kim et al., 2025; Kim G. et al. 2024b; Sjoerdsma et al., 2024). This approach not only preserves the bioactivity of the growth factors but also allows for controlled release over time, which is essential for tissue regeneration.

Another strategy to create Bioinks is delivering growth factors by direct inclusion. Growth factors can be directly mixed with bioinks, although this method requires careful consideration of the printing conditions to maintain their bioactivity. For example, bone morphogenetic protein-2 (BMP-2) has been used in bioinks to stimulate bone growth (Potyondy et al., 2021). The direct inclusion of growth factors can expedite tissue regeneration by providing the necessary biochemical cues to the cells within the printed construct. Similar to growth factors, signaling molecules can be directly incorporated into bioinks to guide cell behavior (Saini et al., 2021). These molecules can include cytokines, chemokines, and other bioactive agents that promote specific cellular responses. The direct inclusion of these molecules ensures that they are uniformly distributed throughout the printed structure, providing a consistent environment for cell growth and differentiation (Saini et al., 2021). Nevertheless, one of the main challenges with direct inclusion is maintaining the bioactivity of the molecules during and after the printing process. Factors like shear stress, temperature, and pH changes during printing can affect the stability of these molecules. Additionally, the release kinetics of directly included molecules can be less controlled compared to encapsulated systems, potentially leading to a burst release that may not be optimal for long-term tissue regeneration.

Recent advances in bioprinting technologies enable direct programming of stem cell-laden bioinks for spatially controlled differentiation, addressing key challenges in tissue complexity and functionality. For instance, the orthogonally induced differentiation (OID) platform uses transcription factor overexpression to bypass media-dependent cues, allowing co-differentiation of hiPSCs into endothelial cells and neurons for vascularized organoids (Skylar-Scott et al., 2022). Another example is the use of photo-crosslinkable ECM bioinks for 3D printing chambered cardiac organoids. These organoids feature a proliferation-first strategy, functional maturation, and perfusable geometry (Kupfer et al., 2020). These technologies overcome traditional limitations in cell density and spatial organization, showing potential for patient-specific models and organ repair. However, challenges remain in scaling cell production and achieving adult-like tissue maturity and size.

##### 4.1.3.2 4D printing

4D printing represents a groundbreaking evolution in additive manufacturing, which integrates time into the design and functionality of printed objects as the “fourth” dimension. This technology uses smart materials that can transform their shape, properties, or functionality when exposed to stimuli like temperature, light, pH, or moisture. These transformations are pre-programmed into the material during the printing process, allowing for dynamic, adaptive structures with applications in personalized medicine, soft robotics, architecture, and textiles. However, challenges in material development, design, scalability, and durability must be addressed to fully realize the potential of 4D printing (Terrovitis et al., 2010; Ashammakhi et al., 2019).

Emerging 4D bioprinting techniques, such as granular support baths that guide post-printing tissue maturation (Pramanick et al., 2025), now enable dynamic shape-morphing constructs that recapitulate developmental processes. These innovations address scalability challenges in scaffold-free systems while preserving ECM-like microenvironments, as seen in Brassard et al.’s bioprinted intestinal crypt-villus units. These units self-organize via Wnt/β-catenin gradients (Brassard et al., 2021). Together, these advances highlight a paradigm shift toward biomanufacturing of implantable tissues with clinically relevant cell densities and functionality.

##### 4.1.3.3 Next-generation bioprinting: Fabrication of functional tissues from stem cells

Traditional methods for 3D bioprinting face a trade-off between printability and a suitable cellular environment, compromising conditions necessary for cell survival, proliferation, differentiation, and the achievement of high cellular density required for complex tissue engineering. Furthermore, they struggle to reliably reproduce physiological tissue-tissue interactions crucial for organ development (Lam et al., 2023). While stem cell-derived organoids are excellent at reproducing local tissue features, they typically cannot be grown beyond the millimeter scale and lack the architectural features of native organs. Previous bioprinting strategies, such as printing cell-only bioink inside support baths or printing biomaterial-based bioink inside self-healing hydrogels, have not provided the necessary environment for complex self-organization (Farsheed et al., 2024; Kelm et al., 2010).

Recent years have witnessed significant advancements in the bioprinting of high-density stem cell-derived tissues. One notable approach involves the use of spheroid fusion within self-healing hydrogels (Daly et al., 2021). This method allows for the creation of heterogeneous tissue models with high cell densities, which is essential for replicating the complex cellular interactions found in native tissues. Additionally, there have been substantial contributions on the development of techniques for biomanufacturing organ-specific tissues with embedded vascular channels, significantly enhancing the scalability and functionality of bioprinted tissues (Skylar-Scott et al., 2019). Moreover, the potential of organoid bioprinting to recapitulate macro-scale tissue self-organization has been successfully demonstrated which is a critical capability for achieving tissue-specific hierarchies and functionalities (Brassard et al., 2021).

The integration of these advanced bioprinting techniques holds significant promise for the development of functional tissue models with tissue-relevant cell densities. By combining strategies such as orthogonally induced differentiation with novel hydrogel matrices and vascularization techniques (Skylar-Scott et al., 2022), researchers can create complex, vascularized organoids that closely mimic native tissue structures and functions. These advancements not only enhance our understanding of tissue development and disease modeling but also pave the way for the creation of clinically viable implants and therapeutic tissue substitutes. As such, they represent a critical step forward in translating bioprinting technologies into practical medical applications.

### 4.2 Small extracellular vesicles

Small extracellular vesicles (sEVs) are nanoscale, lipid bilayer-bound vesicles secreted by cells into the extracellular environment. These vesicles play a pivotal role in intercellular communication by transferring bioactive molecules such as proteins, lipids, mRNAs, and miRNAs between cells. sEVs are generally defined by their size, ranging from 30 to 150 nm in diameter, and their biogenesis, which involves the formation of intraluminal vesicles (ILVs) within multivesicular bodies (MVBs) that are subsequently released into the extracellular space upon fusion with the plasma membrane. This process differentiates sEVs from larger extracellular vesicle subtypes like microvesicles (100–1,000 nm), which bud directly from the plasma membrane (Gao et al., 2021; Nederveen et al., 2021; Jia et al., 2022). The term “exosomes” is often used interchangeably with sEVs; however, according to the Minimal Information for Studies of Extracellular Vesicles (MISEV2018) guidelines, “exosomes” specifically refer to vesicles derived from MVB exocytosis. In contrast, “small extracellular vesicles” is a broader term encompassing all EVs within this size range, regardless of biogenesis. Molecular markers such as CD9, CD63, and CD81 are commonly used to identify sEVs and distinguish them from other EV subtypes (Moerkens et al., 2024; Tang et al., 2022a; Mozneb et al., 2024).

The most prominent application of small extracellular vesicles (sEVs) in personalized medicine is their use as diagnostic markers. The molecular cargo of sEVs, which reflects the physiological or pathological state of their parent cells, makes them invaluable for non-invasive liquid biopsies. These biopsies can identify disease-specific biomarkers, enabling early diagnosis and prognosis. For example, miRNAs and proteins carried by sEVs serve as reliable indicators of tissue health or disease progression (Beetler et al., 2023; Goričar et al., 2021; Maniya et al., 2024).

Beyond diagnostics, sEVs hold great promise as therapeutic delivery systems. Their natural biocompatibility and low immunogenicity compared to synthetic nanoparticles make them ideal carriers for therapeutic agents. sEVs can encapsulate drugs or genetic material and deliver them selectively to target cells, thereby minimizing systemic toxicity and enhancing treatment efficacy (Wang and Pan, 2023; Kumar et al., 2024). Furthermore, one of the most exciting applications of sEVs in stem cell engineering lies in their tailored therapeutic potential. By isolating and analyzing patient-specific sEV profiles from biological fluids, clinicians can identify unique molecular signatures that guide individualized treatment strategies. This approach enables the design of targeted therapies tailored to a patient’s specific needs and allows for the prediction of treatment responses, further advancing precision medicine (Lee et al., 2023; Carnino et al., 2021; Vinik et al., 2020; Tsering et al., 2024).

sEVs also have the ability to create and modulate stem cell niches.

Therefore, embedding stem cell-derived sEVs (SC-sEVs) into biomaterials can be used to develop scaffolds that closely mimic natural stem cell niches. These biomimetic scaffolds can release sEV cargo in a controlled manner, influencing stem cell differentiation and significantly enhancing tissue regeneration (Wu et al., 2022; Re et al., 2021; Swanson et al., 2020). Additionally, sEVs can be engineered to further expand their functionality within the niche. For instance, SC-sEVs can be designed to modulate immune responses by delivering anti-inflammatory molecules or promoting a reparative phenotype in immune cells, creating an environment conducive to healing and regeneration (Kumar et al., 2024; Karnas et al., 2023).

Moreover, it is becoming increasingly reliable to tailor the cargo of stem cell-derived small extracellular vesicles (SC-sEVs) to address patient-specific needs (Tan et al., 2024; Crum et al., 2022). This progress paves the way for the development of not only personalized therapies but also highly effective regenerative treatments. However, while this approach holds great promise, it has not yet been widely explored in the context of SC-sEVs compared to other applications, such as cancer therapy (Kim et al., 2025; Qian et al., 2022; Guo et al., 2025; Joo et al., 2023). These customized approaches address unique pathological conditions while minimizing adverse effects, representing a significant step forward in precision medicine and regenerative therapy.

## 5 Conclusions and future directions

Stem cells, particularly SSCs and iPSCs, are increasingly common in disease modeling and tissue engineering. They model hard-to-access cell varieties, provide a human-based alternative to mouse models for disease research and drug screening, and are used in organoids and tissue constructs. Simultaneously, stem cell based treatments are beginning their journey in the clinical sphere, with hematopoietic and mesenchymal stem cells assisting in the recovery of patients with blood cancers. As new therapeutic approaches emerge, it becomes increasingly crucial to engineer solutions to the concerns commonly associated with stem cells and stem cell treatments, specifically regarding safety and reproducibility. Safety in the context of tissue engineering means ensuring that iPSC and SSC-derived cells are fully differentiated, meaning they will not form unwanted tissues or teratomas, as well as guaranteeing that the cells are functionally mature. Repeatability, on the other hand, means addressing the effects of cell and donor origin on iPSC differentiation, confronting the variability of stem cell derived models in research, and confirming uniformity in treatments derived from stem cell outputs, such as the secretome of MSCs.

One approach to these problems is to use biomaterials that are specifically tailored to stem cells and their desired applications. As more is learned about stem cell behavior *in vivo*, it becomes clear that they are heavily influenced by their surrounding environment. Biomaterials can be designed to provide the mechanical, chemical, and biological signals necessary to control stem cell maintenance and differentiation. This review highlights examples of biomaterials pushing stem cells towards a desired fate, increasing the maturity of derived cells, and increasing the uniformity of stem cell secreted factors.

A bottom-up approach to biomaterials, in which biomaterials are designed with cellular needs as the guiding principle, becomes more feasible when taking into account the variety of biomaterials available and their tunability. Biomaterials can be tailored to specific stiffnesses, given functional groups, and combine natural and synthetic materials. They can further be modified to contain chemical and oxygen gradients. In this way, researchers can improve stem cell maintenance and differentiation by deliberately engineering their environmental cues. A biomaterial-based approach to stem cells is also appropriate when considering their use in engineered tissues. Several biomaterials can act as cellular scaffolds, be 3D printed into complex shapes, or be designed to facilitate tissue self-assembly.

Conventionally, biomaterials for stem cell applications often focus on hydrogels. However, other innovative technologies can bridge stem cell engineering with translational medicine. These include using “backpack molecules” to host cells or the use of hydrogels with an external stimuli to facilitate the production of extracellular vesicles. In theory, a combination of these approaches could be used to deliver specific cargoes to promote cellular responses such as vascularization or tissue regeneration.

For the field of regenerative medicine, stem cells represent a vast, untapped reservoir of potential. Although they have shown promise for tissue repair *in vitro*, few stem cell treatments have reached the clinical setting. The enduring vision of replacing damaged tissues with those grown from a patient’s own cells, or establishing a new route to tissue regeneration, has been hindered by variable stem cell differentiation and the difficulty of replicating complex systems *in vitro*. To bring stem cell products to patients demands a revolutionary change in the approach to differentiate stem cells and how to incorporate them into engineered tissues. Rather than a top-down approach that focuses on replicating specific tissues, we propose a bottom-up approach that focuses on creating biomaterials that support the differentiation and maturation of stem cells, then applying tissue engineering techniques such as 3D printing or self-assembly to confer structure.

Our bottom-up strategy emphasizes the understanding of the intricate cues that influence stem cell behavior, enabling us to craft biomaterials that offer a tailored and adaptable environment. By incorporating engineered elements like sensitive linkages, peptides, growth factors, among other chemical and mechanical cues to the scaffolds and understanding their interaction with the stem cells, we can specifically design biomaterials to meet their needs, enhancing cell viability and scalability. This tailored strategy enhances the safety and efficacy of stem cell therapies by offering a highly controlled and adaptable environment that optimizes cell function, paving the way for their advancement into clinical settings.

## Glossary

ECFCs: Endothelial Colony-Forming Cells
LECs: Lymphatic Endothelial Cells
ECs: Endothelial Cells
ESCs: Embryonic stem cells
iPSCs: Induced Pluripotent Stem Cells
PSCs: Pluripotent Stem Cells
hPSCs: Human Pluripotent Stem Cells
NSCs: Neural Stem Cells
hADSCs: Human adipose-derived stem cells
hMSCs: Human Mesenchymal Stem Cells
hES: Pluripotent human embryonic stem
BMSCs: Bone marrow mesenchymal stem cells
HVSMCs: Human vascular SMCs
hNP: Human neural progenitor
hMSC: Human multipotent mesenchymal stem cell
HUCMSCs: Human umbilical cord blood mesenchymal stem cells
UCB-MNCs: Mononuclear cells of rat umbilical cord blood cells
hASCs: Human adipose stem cells
DPSCs: Human dental pulp stem cells
NIH/3T3: Mouse NIH/Swiss embryo fibroblast
HPC: Hematopoietic stem and progenitor cells
HGFs: Human gingival fibroblasts
HUVECs: Human umbilical vein endothelial cells
hCPCs: Resident cardiac progenitor cells
hDFs: Human somatic dermal fibroblasts
ASCs: Adipose-derived stem cells
hMSC: Human mesenchymal stromal cells
GSCs: Glioma stem-like cells
DPSCs: Dental Pulp Stem Cells
SCCs: Spermatogonial stem cells
ASCs: Adult stem cells
BMDMs: Bone marrow-derived macrophages
BMMs: Bone marrow-derived monocytes
